# On the Luminescence Properties and Surface Passivation Mechanism of III- and N-Polar Nanopillar Ultraviolet Multiple-Quantum-Well Light Emitting Diodes

**DOI:** 10.3390/mi11060572

**Published:** 2020-06-05

**Authors:** Moheb Sheikhi, Yijun Dai, Mei Cui, Liang Li, Jianzhe Liu, Wenan Lan, Rongrong Jiang, Wei Guo, Kuan W.A. Chee, Jichun Ye

**Affiliations:** 1 Ningbo Institute of Materials Technology and Engineering, Chinese Academy of Sciences, Ningbo 315201, China; moheb@nimte.ac.cn (M.S.); daiyijun@nimte.ac.cn (Y.D.); cuimei@nimte.ac.cn (M.C.); lliang@nimte.ac.cn (L.L.); jiangrr@nimte.ac.cn (R.J.); jichun.ye@nimte.ac.cn (J.Y.); 2 University of Chinese Academy of Sciences, Beijing 100049, China; 3 Zhe Jiang Bright Semiconductor Technology Co., Ltd., Jinhua 321016, China; jzliu@bst-group.cn (J.L.); ben@bst-group.cn (W.L.); 4 Hefei National Laboratory for Physical Sciences at Microscale, and Department of Physics, University of Science and Technology of China, Hefei 230026, China; 5 Laser Research Institute, Shandong Academy of Sciences, Qingdao 226100, China

**Keywords:** III-nitride thin film, nanostructures, ultraviolet emitters, surface passivation, luminescence intensity

## Abstract

The non-centrosymmetricity of III-nitride wurtzite crystals enables metal or nitrogen polarity with dramatically different surface energies and optical properties. In this work, III-polar and N-polar nanostructured ultraviolet multiple quantum wells (UV-MQWs) were fabricated by nanosphere lithography and reactive ion etching. The influence of KOH etching and rapid thermal annealing treatments on the luminescence behaviors were carefully investigated, showing a maximum enhancement factor of 2.4 in emission intensity for III-polar nanopillars, but no significant improvement for N-polar nanopillars. The discrepancy in optical behaviors between III- and N-polar nanopillar MQWs stems from carrier localization in III-polar surface, as indium compositional inhomogeneity is discovered by cathodoluminescence mapping, and a defect-insensitive emission property is observed. Therefore, non-radiative recombination centers such as threading dislocations or point defects are unlikely to influence the optical property even after post-fabrication surface treatment. This work lays solid foundation for future study on the effects of surface treatment on III- and N-polar nanostructured light-emitting-diodes and provides a promising route for the design of nanostructure photonic devices.

## 1. Introduction

III-nitride based ultraviolet light-emitting-diodes (UV-LEDs) are useful for many applications including UV curing, photo therapy and UV disinfection, due to the direct bandgap property of wurtzite phase III-nitride crystals [1,2,3,4]. However, the extraction efficiency of UV LEDs is relatively low due to the strong internal light reflection and strong transverse magnetic (TM) polarization of the emitted light [5,6,7]. Challenges in light extraction have encouraged research and development of small length scale devices for new applications, like nanostructured LEDs, for example [8,9,10]. The three-dimensional geometry of the nanopillars or nanoholes allows light to be extracted from the sidewalls of the nanostructures. This becomes increasingly important as the emission wavelength of the LEDs moves from visible to ultraviolet wavelength region due to increasing proportion of TM-polarized light, as well as the growing impact of light absorption in the p-AlGaN contact layer [11,12]. Additionally, the benefits from strain relaxation in nanostructured LEDs means reduced influence from quantum confined stark effect (QCSE), which has an adverse impact on the electron and hole wavefunction overlap [13].

Currently, most of the nanostructured UV-LEDs are fabricated by reactive ion etching (RIE) due to better control over morphologies and sizes of the nanostructures [14]. However, RIE introduces surface defect states that will lead to reduced external quantum efficiency (EQE) and degraded output power of LEDs through Shockley–Read–Hall (SRH) non-radiative recombination [15]. The large surface areas of GaN nanopillars can lead to dangling bonds during nanopillar formation. However, the resulting surface defects can be reduced by passivation techniques such as chemical etching or dielectric deposition [16]. It was reported that, plasma-related damages at the sidewalls of multiple quantum wells (MQWs) induced by RIE can be readily healed by thermal annealing, reducing the density of surface traps [17]. Therefore, eliminating the surface defect states is strongly required [18]. Chiu et al. reported that after a photo-enhanced chemical (PEC) wet oxidation process, emission from InGaN/GaN-based random nanorod LEDs was significantly improved, but also with a 10.5-nm blueshift with respect to the as-grown LED [19]. Sun et al. also reported a 50% enhancement in the UV light emission intensity from the KOH treated InGaN nanowires (NWs) due to the removal of surface dangling bonds [18]. Despite the above promising results, the knowledge regards to surface passivation of nanostructured LED is still not complete, since the majority of the samples are III-polar for better crystalline quality and smoother surfaces. However, N-polar LEDs have their unique advantage such as lower contact resistance, increased current injection efficiency, and an internal polarization field, which is opposite to the external bias, leading to reduced QCSE and higher radiative recombination rate [20,21]. Unfortunately, there seldom are comparative investigations on the effects of surface treatments on the optical properties of both III- and N-polar nanostructured MQWs LEDs, which serve as building blocks of next-generation high-efficiency UV emitters.

In this work, large-scale, highly periodic nanopillar UV MQWs with III- and N-polarities were fabricated by nanosphere lithography and RIE patterning. Chemical treatment and thermal annealing are acknowledged as promising methods to enhance the luminescence property of III-polar nanostructured MQWs by 2.4 times at most, but was demonstrated to show little effect on N-polar nanostructures. The underlying reason for this discrepancy was thoroughly discuss, which can be mainly attributed to intrinsic indium localization and herein defect-insensitive emissions from the N-polar nanostructures. A comprehensive investigation on the surface passivation mechanism on both III- and N-polar nanostructure emitters were provided, benefiting future development of novel nanostructured light emitters.

## 2. Materials and Methods

InGaN/GaN based UV-MQW was grown on 2-inch c-plane sapphire substrate via a low-pressure, high-temperature metalorganic chemical vapor deposition (MOCVD) system. Trimethylindium (TMIn), triethylgallium (TEGa) and ammonia (NH_3_) were used as precursors of In, Ga and N, respectively. Hydrogen (H_2_) was used as the carrier gas. To investigate the polarity influence on the luminescence property of nanopillars, both III- and N-polar planar MQWs were firstly grown. The III-nitride polarity of the MQWs was controlled by the modulation of low-temperature (LT) AlN nucleation layer (NL) prior to high-temperature (HT) epitaxial growth. Generally, III-nitride thin film is III-polarity if it is grown on LT AlN-NL. In contrast, when grown on bare sapphire substrate with proper H_2_ annealing and NH_3_ nitridation condition, the thin film is N-polarity. UV-MQW consists of a 3.5 µm GaN epitaxial layer, eight pairs of In_0.03_Ga_0.97_N/GaN MQWs followed by a 10 nm GaN cap layer. To fabricate nanostructured UV-MQWs, III-polar and N-polar epitaxial thin films are uniformly coated with polystyrene (PS) spheres of 2 µm diameter via a large-area micro-propulsive injection method. The colloidal solution was injected into the water to form a Langmuir–Blodgett film in a hexagonal configuration at the air/water interface. By draining away the water, the monolayer of closely packed PS spheres is transferred onto the MQW thin film. Nanopillar arrays were then achieved by RIE using Cl_2_/BCl_3_ gases to pattern transfer the PS spheres to the underlying MQWs. The remaining PS spheres were removed by sonication in acetone solution. After fabrication of the nanopillar arrays, chemical and thermal treatments were applied to the MQW samples in order to heal the plasma-related damages. For chemical treatment, samples were dipped into KOH aqueous solution with a concentration of 10 wt%. The treatment was performed under room temperature (RT) or 45 °C for 40 s. For thermal treatment, rapid thermal annealing (RTA) was performed at 800 °C or 900 °C for 15 min under N_2_ atmosphere. Surface morphologies of the nanostructures were characterized in the Hitachi S-4800 field-emission (FE) SEM (Hitachi, Tokyo, Japan). Dislocation densities of both samples were characterized using a point-focused high-resolution X-ray (Cu Kα1) diffractometer (HRXRD, Bruker D8 Discover, Germany) equipped with a four-bounce symmetric Ge (220) monochromator. The surface morphology of the MQW structure was characterized by atomic force microscopy (AFM) (Veeco Dimension 3100 V, Plainview, NY, USA). High-angle annular dark field scanning transmission electron microscopy (HAADF-STEM) was performed and weak beam dark field images were acquired under two beam conditions at an acceleration voltage of 300 kV (FEI Titan ST microscope, Hillsboro, OR, USA). Specimens were prepared by focus ion beam (FIB) using an FEI Helios SEM system with a Ga ion source. RT photoluminescence (PL) was performed by using an Ar-F (193 nm) excimer laser (Coherent Inc., Santa Clara, CA, USA) as an excitation pumping source, and the spectra were collected by a Horiba iHR550 spectrometer (Horiba, Kyoto, Japan). Cathodoluminescence (CL) investigations were undertaken using a Horiba MP 325 CL characterization system (Horiba, Kyoto, Japan) with voltage of 5 kV and current level of 188 μA. Strain conditions of the nanopillar MQWs were investigated by a Renishaw inVia Reflex spectrometer system (Renishaw, New Mills, UK) with a 532 nm Nd-YAG laser as the excitation source. The surface stoichiometry of the samples was further studied by Kratos Axis Ultra DLD X-ray photoelectron spectrometer (XPS; Kratos, Manchester, UK).

## 3. Results and Discussion

**Figure 1:**
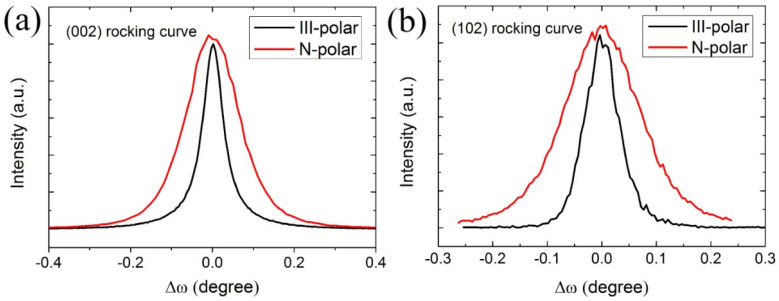
HRXRD (002) (**a**) and (102) (**b**) RC scans of planar III- and N-polar samples for dislocation estimation.

Before detailed investigations on optical properties of nanopillar samples, the structural and surface morphology information of planar III-polar and N-polar MQW samples are investigated. Figure 1 shows the HRXRD rocking curve (RC) scans of both III- and N-polar samples. The full-width-half-maximum (FWHM) values of RC scans were used to evaluate the crystalline quality of III-nitride thin films. From the fitting, the FWHM of (002) and (102) peaks of III-polar sample are 265 and 275 arcsec, respectively, which are lower than those of N-polar samples (586 and 578 arcsec for (002) and (102) peaks). The dislocation density can herein be estimated. Screw and edge type dislocation densities are 1.53 × 10^8^ cm^−2^ and 4.59 × 10^8^ cm^−2^ for III-polar samples, which are approximately 5 times lower than those of N-polar ones, whose values are 7.48 × 10^8^ cm^−2^ and 1.8 × 10^9^ cm^−2^. This result suggests that III-polar samples exhibit superior crystalline quality than N-polar samples.

**Figure 2:**
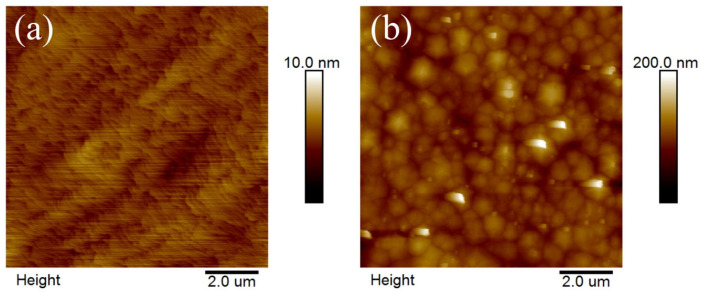
AFM surface morphology of planar III-polar (**a**) and N-polar (**b**) MQW samples.

Surface morphologies of both samples were analyzed by AFM and illustrated in Figure 2. Clear bi-layer steps are illustrated in the III-polar sample, demonstrating a typical step-flow growth mode of III-polar III-nitride thin film [22]. On the other hand, N-polar sample is covered with hillocks of a few micrometers lateral size, suggesting a three-dimensional growth mode because of its much smaller surface energy and thus larger nucleation density [23]. Root-mean-square roughness for III- and N-polar samples are 0.37 and 11.0 nm, respectively. The drastic difference of surface roughness of these two polarities are also correlated with their crystalline quality. Indium content of planar MQW samples can usually be identified from symmetric and asymmetric ω-2θ scans from HRXRD. Symmetric ω-2θ scan of planar III- and N-polar samples was performed and shown in supplementary Figure S1. The most prominent peak can be identified as GaN template. Different peak positions originate from various strain states inside epitaxial thin films as a consequence of growth mode difference between III- and N-polar domains [21]. Usually, 0st order InGaN MQW peak can be found on the low-angle side of the spectrum, but can hardly be found in our study. This could possibly due to the extremely low in content of only 3% in the MQW region and thus MQW peak merges with the GaN template peak.

**Figure 3:**
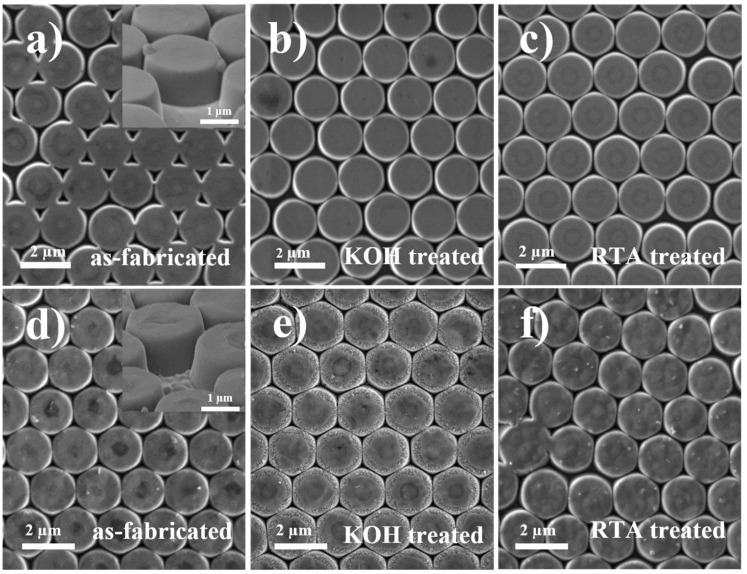
SEM images of III-polar (**a**–**c**) and N-polar (**d**–**f**) nanopillar multiple quantum wells (MQWs) before and after the KOH or rapid thermal annealing (RTA) treatment. Tilted-view SEM images of as-fabricated nanopillars are shown in the inset of (**a**,**d**).

RIE followed by nanosphere lithography was utilized to fabricate the nanopillar UV-MQW samples. The lattice constant of the nanopillar array is expected to match the 2 µm diameter of the PS spheres used in the nanosphere lithography. Due to ion bombardment, amorphous layers are generated on the sidewall surfaces of the nanopillars during RIE patterning, but which are expected to be removed by the KOH treatment. The SEM images in Figure 3 show the surface morphology of the uniformly distributed nanopillar hexagonal array on the as-fabricated III- and N-polar UV-MQWs after subjecting to the different treatments. It can be seen that the diameter of the nanopillars remained the same after RTA but reduced slightly to 1.98 µm after KOH treatment. Thermal annealing does not alter the surface morphologies of the nanopillars due to a lacking of energy for thin film re-crystallization or surface reconstruction. It is known that the dangling bond density for N-polar planes is higher than that of (0001) planes, leading to a much higher etch rate under KOH treatment. Therefore, elongated time of KOH etching could damage the nanopillars as shown in Figure 3e where the flat top mesa of the nanopillars is covered with small hillocks. The 60° tilted view SEM images of the as-fabricated nanopillars are shown in the inset of Figure 3a,d. After RIE process, the depths of the III and N-polar nanopillars are 0.744 and 0.748 µm, respectively, suggesting almost same RIE etching rate between these two polarities.

RT photoluminescence (PL) measurements were carried out to determine the luminescence efficiency of the nanopillar MQWs before and after KOH or RTA treatment. First of all, PL intensities of III- and N-polar nanostructured samples are compared. III-polar nanopillars show stronger intensity than that of N-polar samples. This could because of two reasons: firstly, a strong carrier localization effect is observed in N-polar nanopillar sample as will be demonstrated later. This could originate from rough surface morphology of N-polar planar sample to begin with, and MQW thickness fluctuations as demonstrated in our previous work [20]. Even though carrier localization can greatly enhance internal quantum efficiency in LEDs, but excessive phase separation can also lead to a greatly reduced active area of the MQWs at the same time, which is detrimental to the optical property as observed in this work. Secondly, N-polar sample exhibits higher dislocations than that of III-polar samples. This greatly deteriorate the luminescence intensity of N-polar nanostructured samples. PL spectra of planar III- and N-polar MQW samples are shown in supplementary Figure S2 for comparison purpose. No obvious variation in peak position is observed between planar and nanostructured sample. However, an enhanced luminescence intensity is identified for the nanostructured sample due to enhanced light scattering effect [14].

**Figure 4:**
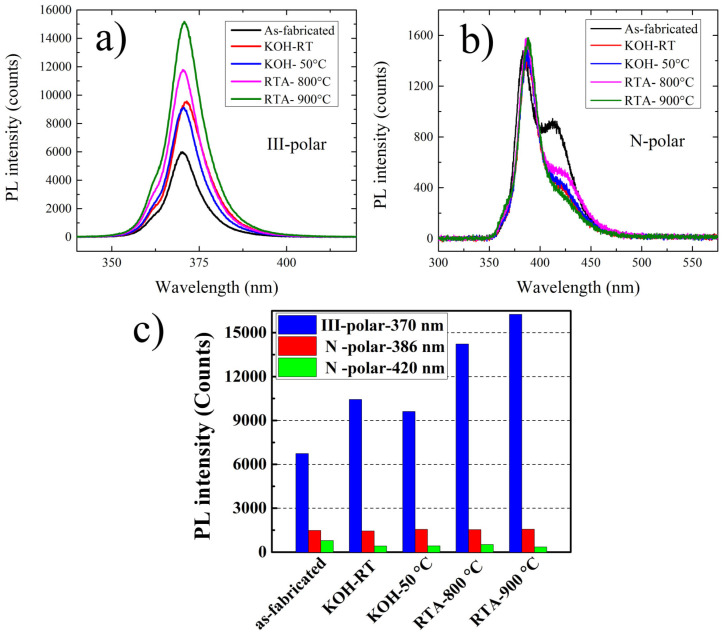
Room temperature (RT) photoluminescence (PL) spectra of III-polar (**a**) and N-polar (**b**) nanopillar MQWs, and integrated PL intensity of III-polar and N-polar MQW nanopillar MQWs at the specific peak wavelengths (**c**), before and after KOH treatment or rapid thermal annealing (RTA).

PL spectra subject to different surface treatments were further investigated. Figure 4a,b shows the PL spectra of III- and N-polar nanopillar MQWs after the respective treatments. PL intensities dramatically increased for III-polar compared to N-polar nanopillar MQWs. The highest PL enhancement reached 2.4 times for RTA of 900 °C. This can be well explained by the passivation of surface traps and the healing of surface-related defects [14]. However, the luminescence spectra were dramatically different for N-polar nanopillar MQWs. For the N-polar nanopillar MQW, a peak and a broad shoulder were identified in the PL spectra where the left peak was located at approximately 386 nm, and the right shoulder was located at 420 nm. The left peak intensity remained roughly the same after KOH or RTA treatment, while the longer wavelength shoulder reduced, suggesting that polarity played a critical role in the influence of surface treatment on luminescence. The integrated PL intensities of the III- and N-polar nanopillars are shown in Figure 4c, indicating that the effect of surface treatment was marginal on the N-polar MQWs compared to that of the III-polar samples.

The strong variation of PL peak position from III- and N-polar samples is originated from either higher indium composition or thickness variation or a combination of both. An average indium content changing from 1.3% to 5.2% in the MQW is expected when composition is considered as the only factor. For the 420 nm shoulder peak in N-polar MQW sample, a further increase of indium content to 12.6% is obtained. The optical transition in the QWs was further analyzed by solving the Poisson equations and carrier transport equations. During calculation, 3/11 nm In_0.03_Ga_0.97_N QW and GaN QB thicknesses are utilized. As shown in the calculation result illustrated in supplementary Figure S3, a broad emission peak with position located at 365 nm is identified, which is slightly shorter than the 370 nm emission peak observed in PL spectra. The internal electric field inside QW is less than 1 × 10^5^ V/cm, which is relatively low compared to AlGaN or InGaN system reported elsewhere [24,25]. The discrepancy between the simulated electroluminescence (EL) spectrum and experimental PL spectrum can be explained by a variety of factors including different interaction volumes between injected current in EL and excited photons in PL, deviation of indium composition from target and QW thickness fluctuation. But we can safely conclude that the uniform emission peak from III-polar sample is a consequence of efficient radiative recombination inside the abrupt QWs as shown from STEM image of III-polar MQW sample observed in supplementary Figure S4, while non-uniform emission in N-polar sample is a result of carrier localization.

Finally, in order to correlate PL spectra with the influence of QW thickness variation, emission spectra are simulated based on an LED with 3 pairs of MQWs. The indium content in QW is fixed at 3%, while QW thickness was varied from 3 nm to 6 nm. The thickness of quantum barrier remains constant at 11 nm. From the simulation results shown in supplementary Figure S5, two emission peaks can be identified. This occurrence of two peaks can be explained by the shallow QW and thus weak carrier confinement. The high-energy emission peak approximately locates at 364 nm, and does not change with QW thickness. On the other hand, the low-energy peak red shifts as increasing QW thickness, in good agreement with the reduced quantum confinement and thus lower emission energy [26]. However, the red shift is only less than 7 nm, which is far less than that observed in the experiment. The coherence with a 420-nm emission cannot be simulated by only changing the QW thickness, which further demonstrates that composition inhomogeneity must play an important role in the light emission.

**Figure 5:**
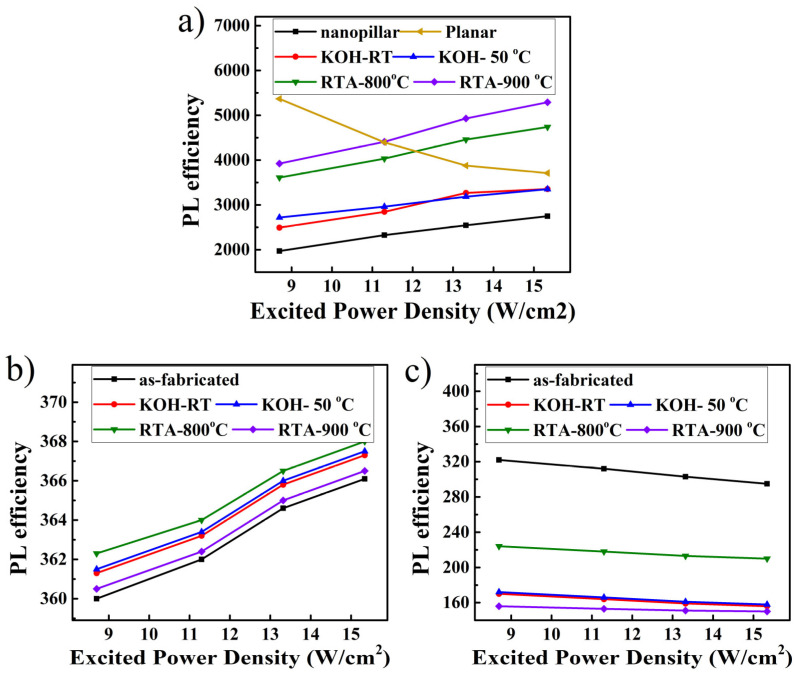
PL efficiency as a function of excited power density for III-polar nanopillar MQWs (**a**), and N-polar nanopillar MQWs with emission wavelength located at 386 nm (**b**) and at 420 nm (**c**) respectively.

**Figure 6:**
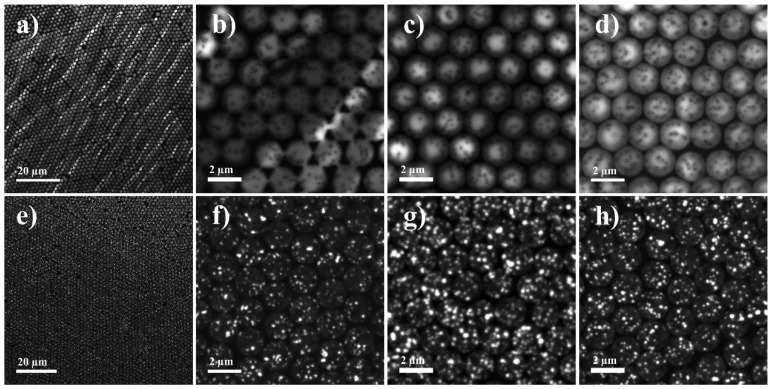
Low magnification (**a**) and high magnification panchromatic cathodoluminescence (CL) mapping of as-fabricated (**b**), KOH treated (**c**) and thermally treated (**d**) III-polar nanopillar MQWs. CL mapping of N-polar nanopillar MQWs are illustrated in (**e**–**h**), accordingly.

**Figure 7:**
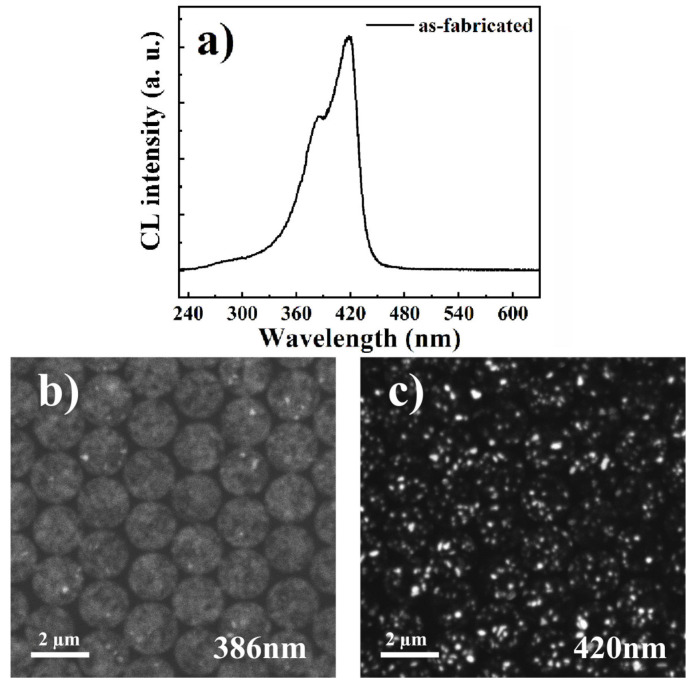
CL spectrum of N-polar nanopillar MQW illustrating two peaks at 386 and 420 nm, respectively (**a**). Monochromatic CL intensity distribution of N-polar nanopillar MQW at 386 nm (**b**) and 420 nm (**c**), respectively.

To further understand the PL enhancement mechanism of III-polar nanopillar MQWs, PL efficiency (PL_eff_) as a function of excited power density of the III and N-polar MQW samples is illustrated in Figure 5. PL_eff_ is defined as the ratio of PL intensity to excited power density, and its slope is an indication of radiative recombination mechanism [10]. The negative slope of the PL_eff_ versus excited power density for planar MQWs suggests the existence of exciton-related emission together with large amount of non-radiative centers. In contrast, for III-polar nanopillar samples, the PL_eff_ increased when the pumping power density increased, which is a signature of radiative carrier recombination through free carriers. The PL_eff_ increased for KOH treatment at a higher temperature. Higher PL_eff_ was obtained after thermal annealing. The slightly larger slope of PL_eff_ vs. pumping power density after thermal annealing shows an enhanced radiative recombination rate, which might indicate annihilation of trap defects. For N-polar nanopillar MQWs, since the emission spectra had two peaks locating at 386 nm and 420 nm, thus two PL_eff_ curves are illustrated. For a 386 nm emission in N-polar MQW, PL_eff_ also increased with pumping power density similar to that of III-polar nanopillars. However, a negative slope was observed for N-polar nanopillars with 420 nm emission, again suggesting the exciton-related emission mechanism, which was a signature of carrier localization effect as will be discussed in details later in Figure 6 and Figure 7.

Due to phase separation between In and Ga, compositional inhomogeneities in InGaN alloys lead to non-uniform optical properties of UV-LEDs, especially for N-polar surfaces where rough surface morphology promotes phase separation. Therefore, deeper understanding on the spatially resolved luminescence properties of III- and N-polar nanopillar MQWs is critically important. Panchromatic CL intensity mapping is shown in Figure 6. Figure 6a–d illustrates the CL distribution in III-polar nanopillar MQWs and Figure 6e–h illustrates the CL distribution in N-polar nanopillar MQWs. Under low magnification, bright stripes along the substrate offcut direction were clearly observed on the surface of the III-polar nanopillar MQW (Figure 6a), which could be attributed to intrinsic indium localization in the MQWs. The strong luminescence comes from the In-rich clusters along the step edges of the epitaxial thin films. Since white stripes are shown in both planar and nanostructured MQWs, nanopillar fabrication did not have an influence on the distribution of luminescence centers. A closer look at the spatially resolved CL intensity distribution of III-polar MQWs in Figure 6b–d gives us clear information that luminescence only coming from the nanopillars where MQWs were not etched away by RIE. Additionally, the emission intensity increased dramatically after thermal annealing of 900 °C, which agreed perfectly with the enhanced PL intensity as shown before in Figure 4. Different from that of III-polar samples, strong localization of luminescence centers is shown for all N-polar samples. The dot-like bright features have lateral size of 20–200 nm. White dots represent quantum-dot-like luminescence centers, which were uniformly distributed on the sample, possibly due to the rough surface morphology of N-polar MQWs and thus non-uniform light emission. Note that carrier localization is also observed in planar samples. This has been thoroughly discussed in our previous work [27,28]. Carrier localization was observed in N-polar domain of planar sample due to thickness and composition fluctuation. However, carrier localization is not observed in III-polar MQW sample. This is due to the fact that the indium composition is too low for spinodal decomposition. In fact, carrier localization was widely reported in InGaN with high indium content above 15% [29], but seldom been reported in UV-LEDs with InGaN MQWs of indium content less than 5% as studied here.

Additionally, the black dots were identified throughout the surface in the panchromatic CL intensity mapping shown in Figure 6b–d, representing non-radiative recombination centers like threading dislocations or point defects. The densities were 2.35 × 10^8^ cm^2^, 2.29 × 10^8^ cm^2^ and 2.38 × 10^8^ cm^2^ respectively for as-fabricated nanopillars, nanopillars subject to KOH treatment and thermal annealing, respectively. No obvious change in the defect density after thermal or KOH treatment was identified, suggesting that luminescence enhancement as shown in Figure 4 is mainly related to passivation of surface states, rather than defects in the bulk.

Figure 7a shows the CL spectrum of the as-fabricated N-polar nanopillars with two peaks located at 386 and 420 nm. The monochromatic CL mapping at 386 nm and 420 nm are shown in Figure 7b,c, respectively. Interestingly, the CL emission at 386 nm was uniformly distributed on the surface of the nanopillars, whereas 420 nm-emission originated from In-rich clusters, which are represented by the white dots. The red shift of CL spectra for N-polar nanopillars compared to that of the III-polar sample can be well explained by the in-phase separation in the MQWs leading to quantum dot-like luminescence centers at those In-rich clusters. Additionally, the carrier diffusion length decreased drastically due to indium phase separation, and the influence of point defects and threading dislocations were mostly screened by these potential minima, imposing no effect on the luminescence intensity. Therefore, even though surface trap defects were passivated by thermal and chemical treatments the luminescence intensities still remained the same for N-polar nanopillar MQWs because of carrier localization effects.

**Figure 8:**
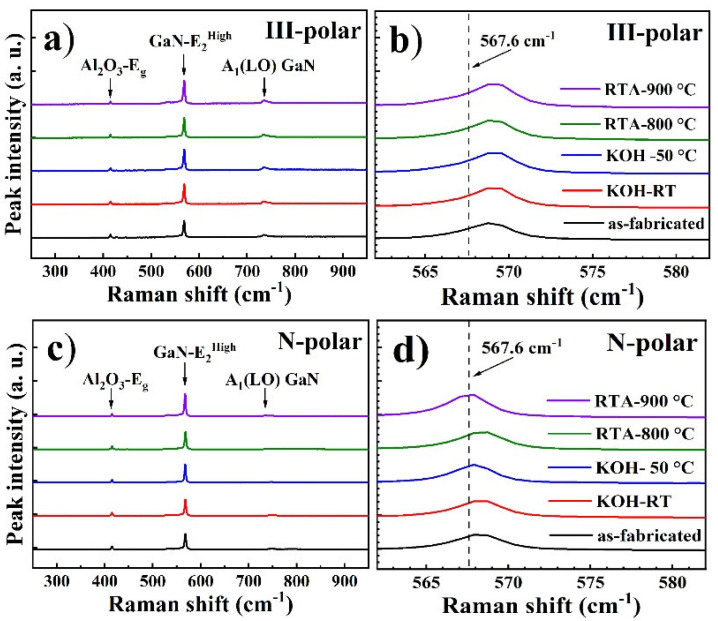
Full-range (**a**,**c**) and zoom-in (**b**,**d**) Raman spectra of III-polar (**a**,**b**) and N-polar (**c**,**d**) nanopillar MQWs before and after chemical and thermal treatments.

Optical properties of MQWs were also correlated to strain conditions. Therefore, Raman spectroscopy investigations were performed. Figure 8 shows the Raman spectra recorded for III- and N-polar nanopillars before and after RTA or KOH treatment. The peak position of the E_2_ (high) phonon mode of III-nitride crystal is sensitive to bi-axial strains [30]. For fully relaxed GaN, the E_2_ (high) peak was at 567.6 cm^−1^, and a smaller Raman wavenumber suggests tensile strain while a larger Raman wavenumber suggests the compressive strain. In this work, since the epitaxial thin film was mainly composed of GaN or AlGaN with low Al composition, E_2_ (high) peak located around 567 cm^−1^ was regarded as fully-relaxed, and was utilized for strain analysis. The peak positions for all the III-polar nanopillar MQW samples were close to each other with a value of 569 cm^−1^ indicating slightly compressive strains of 0.6 Gpa regardless of chemical or thermal treatment. Note that the E_2_ (high) position of the as-fabricated planar MQW was located at a much high wavenumber of 572.1 cm^−1^ as observed in our recent study. Therefore, most of the strains were actually relaxed after nanopillar fabrication. Compared to the III-polar nanopillar samples, the compressive strain in as-fabricated N-polar nanopillar samples was found to be much smaller as the E_2_ (high) position of the GaN-like Raman peak was only 1 cm^−1^ higher at maximum compared to the fully relaxed position. This could be explained by the 3D growth mode of N-polar thin film, higher dislocation density and thus more strain relaxation inside N-polar crystals. RTA treatment at 900 °C fully relaxes the compressive strain inside the as-fabricated N-polar nanopillars, even though no significant improvement in emission intensity was found.

**Figure 9:**
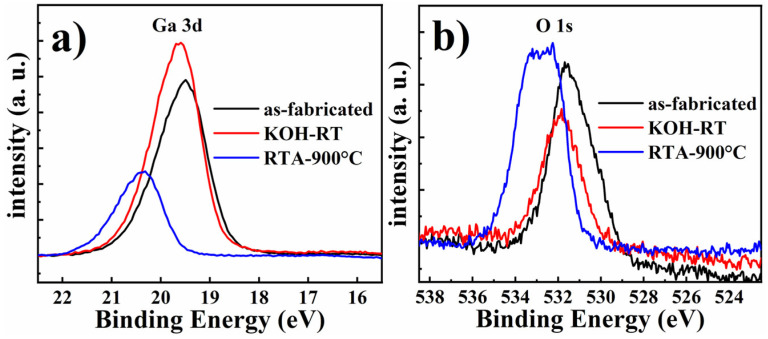
High resolution Ga 3d (**a**) and O 1s (**b**) XPS core level spectra of III-polar nanopillar MQWs without treatment and after KOH or RTA treatments.

Finally, to further correlate surface stoichiometry to the enhanced UV emission of III-polar nanopillar MQWs, XPS measurements were carried out before and after RTA or KOH treatment. The de-convoluted Ga 3d peaks of the as-fabricated nanopillars, nanopillars after KOH treatment and 900 °C annealing are shown in Figure 9a. As indicated in the figure, the major Ga 3d core level peak consisted of two components at 21.0 ± 0.1 eV and 19.3 ± 0.1 eV corresponding to the binding energy of Ga–O and Ga–N, respectively. After KOH etching, the Ga 3d peak slightly shifted to higher binding energy, where the relative Ga–O component was increased, indicating that Ga cations were attacked by OH–, leading to the breakage of Ga–N bond and consequently formation of Ga–O bond. Furthermore, after RTA surface treatment, the Ga 3d peak shifts to even higher binding energy, but the total intensity was dramatically reduced. This indicates that chemical stoichiometry between Ga and N atom was deviating from 1:1 after thermal annealing. Figure 9b shows the O 1s core level peaks de-convoluted into three components with binding energies of 530.3 eV, 531.7 eV and 532.8 eV, corresponding to O^2−^, OH^−^ and H_2_O species, respectively. It is clearly shown that after KOH etching, the overall intensity of the O 1s peak decreased, in agreement with the removal of naturally formed surface oxide under KOH etching. After thermal annealing, there existed a strong shift of O 1s peak towards higher binding energy, indicating a transformation from the Ga–O bond to Ga–O–H bond.

## 4. Conclusions

In this work, the influences of KOH or RTA treatments on the optical properties of III and N-polar nanopillar UV MQWs were carefully examined. The UV emission intensity increased significantly after post-fabrication treatment and by as much as 2.4 times after RTA annealing for III-polar nanopillars, whereas almost no effect on the luminescence spectra was seen for N-polar samples. CL intensity maps indicate carrier localization effects due to In-rich clusters in the N-polar nanopillars, which dominated the light output mechanism because of the reduced carrier diffusion length scales. This led to reduced probability of carrier recombination with threading dislocations or point defects, meaning that despite the fact that surface treatment passivation methods might reduce the trap defects, they did not contribute to the PL efficiency. Slight compressive strain effects could be found in as-fabricated III-polar nanopillars, but which did not change significantly after KOH or RTA treatment. As-fabricated N-polar nanopillar samples were almost strain-free due to 3D growth mode and strain relaxation mechanism. XPS results further suggest the removal of surface oxides after KOH etching and transformation from Ga–O to Ga–O–H bond after thermal annealing for III-polar nanopillar samples. The experimental results obtained here present a deeper understanding on the different optical behaviors of nanostructured UV emitters with opposite polarities, and provide an important strategy to enhance the luminescence intensity of nanopillar LEDs, paving the way for future development of novel nanostructure-based electronic or photonic devices.

## Supplementary Materials

The following are available online at https://www.mdpi.com/2072-666X/11/6/572/s1, Figure S1: HRXRD symmetric ω-2θ scans of III- and N-polar MQW samples; Figure S2: RT PL spectra of planar III-polar and N-polar MQW samples. Peak positions and shapes are almost identical to those of nanostructured samples; Figure S3: Simulated electroluminescence spectrum of UVA-LED with 3/11 nm In_0.03_Ga_0.97_N/GaN MQW; Figure S4: HAADF-STEM image of planar III-polar MQW samples with low magnification (**a**) and zoom-in view (**b**) of the individual QW and QB; Figure S5: Simulated electroluminescence spectra of UVA-LED with In_0.03_Ga_0.97_N/GaN MQWs of varying QW thicknesses. A small red shift of less than 7 nm is observed when doubling the QW thickness.
