# Supplementary material for: On the Luminescence Properties and Surface Passivation Mechanism of III- and N-Polar Nanopillar Ultraviolet Multiple-Quantum-Well Light Emitting Diodes
Source: Micromachines (Basel). 2020 Jun 5;11(6):572. doi: 10.3390/mi11060572 (PMC7345201; doi:10.3390/mi11060572)
Supplement: Supplementary file 1 [file micromachines-11-00572-s001.pdf]

Supplementary Materials

On the Luminescence Properties and Surface Passivation Mechanism of III- and N-Polar Nanopillar Ultraviolet Multiple-Quantum-Well Light Emitting Diodes

Moheb Sheikhi [1,2,†], Yijun Dai [1,2,†], Mei Cui [1,2], Liang Li [1,2], Jianzhe Liu [3], Wenan Lan [3], Rongrong Jiang [1], Wei Guo [1,2,*], Kuan W.A. Chee [4,5,*] and Jichun Ye [1,2]

[1] Ningbo Institute of Materials Technology and Engineering, Chinese Academy of Sciences, Ningbo 315201, China; moheb@nimte.ac.cn (M.S.); daiyijun@nimte.ac.cn (Y.D.); cuimei@nimte.ac.cn(M.C.); lliang@nimte.ac.cn (L.L.); jiangrr@nimte.ac.cn (R.J.); jichun.ye@nimte.ac.cn (J.Y.)

[2] University of Chinese Academy of Sciences, Beijing 100049, China

[3] Zhe Jiang Bright Semiconductor Technology Co., Ltd., Jinhua 321016, China; jzliu@bst-group.cn (J.L.); ben@bst-group.cn (W.L.)

[4] Hefei National Laboratory for Physical Sciences at Microscale, and Department of Physics, University of Science and Technology of China, Hefei 230026, China

[5] Laser Research Institute, Shandong Academy of Sciences, Qingdao 226100, China

* Correspondence: guowei@nimte.ac.cn (W.G.); kuan.chee@cantab.net (K.W.A.C.)

† These authors contribute equally to this work.

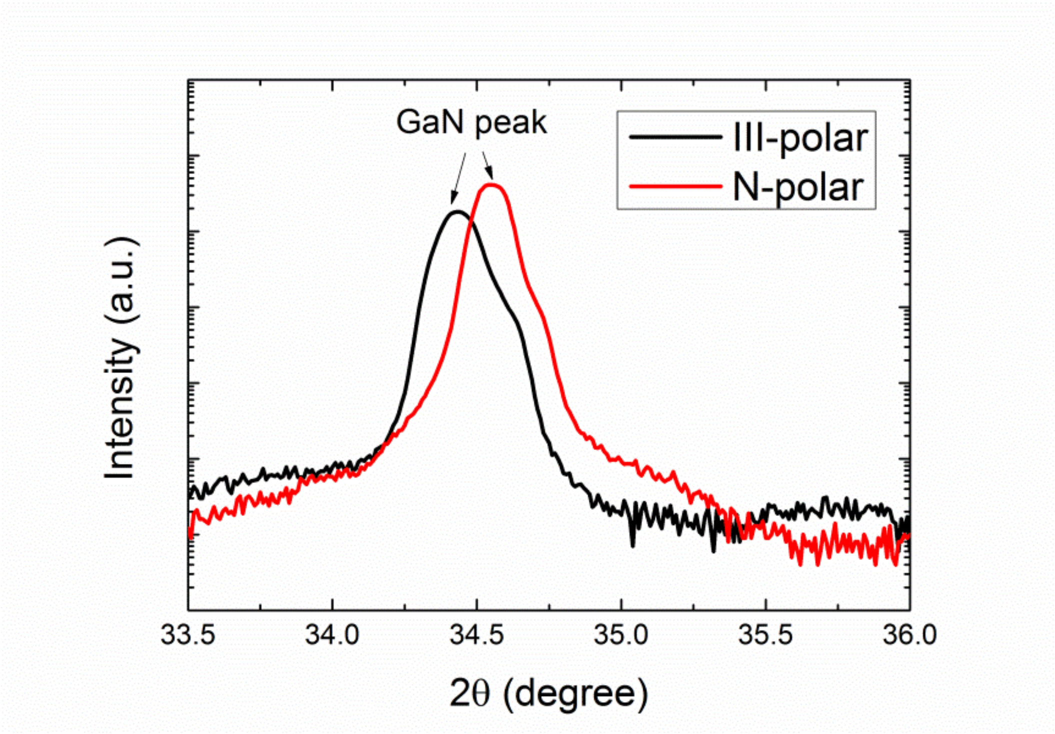

Figure S1. HRXRD symmetric ω-2θ scans of III- and N-polar MQW samples.

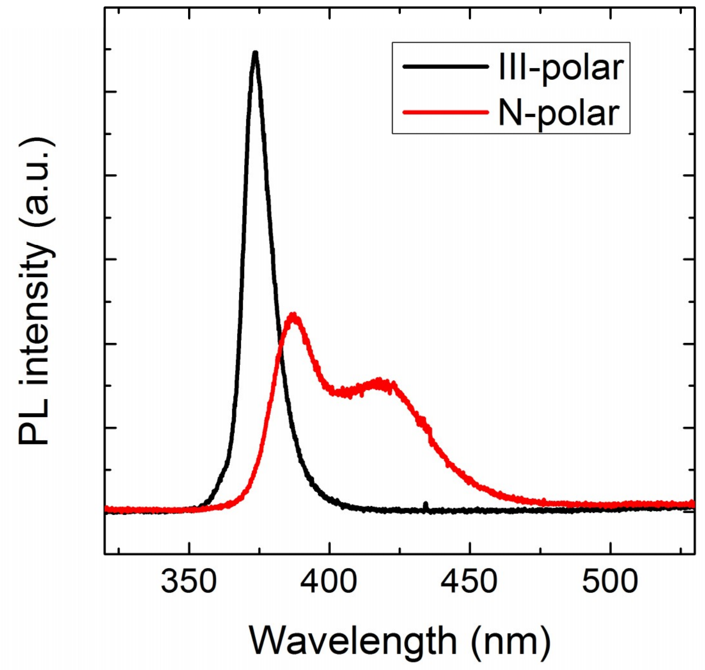

Figure S2. RT PL spectra of planar III-polar and N-polar MQW samples. Peak positions and shapes are almost identical to those of nanostructured samples.

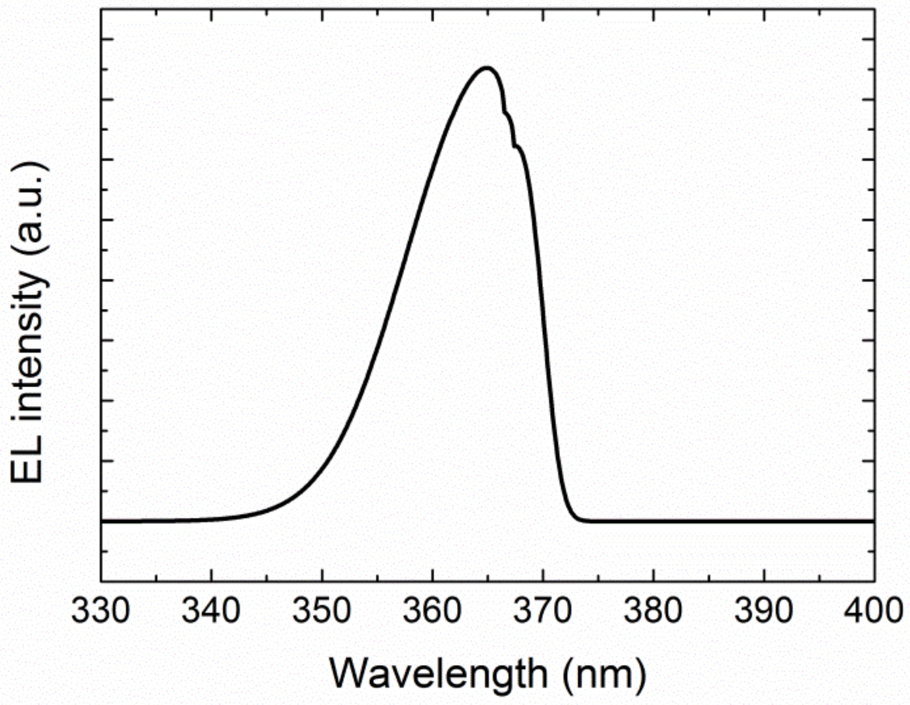

Figure S3. Simulated electroluminescence spectrum of UVA-LED with 3/11 nm $In_{0.03}Ga_{0.97}N$/GaN MQW.

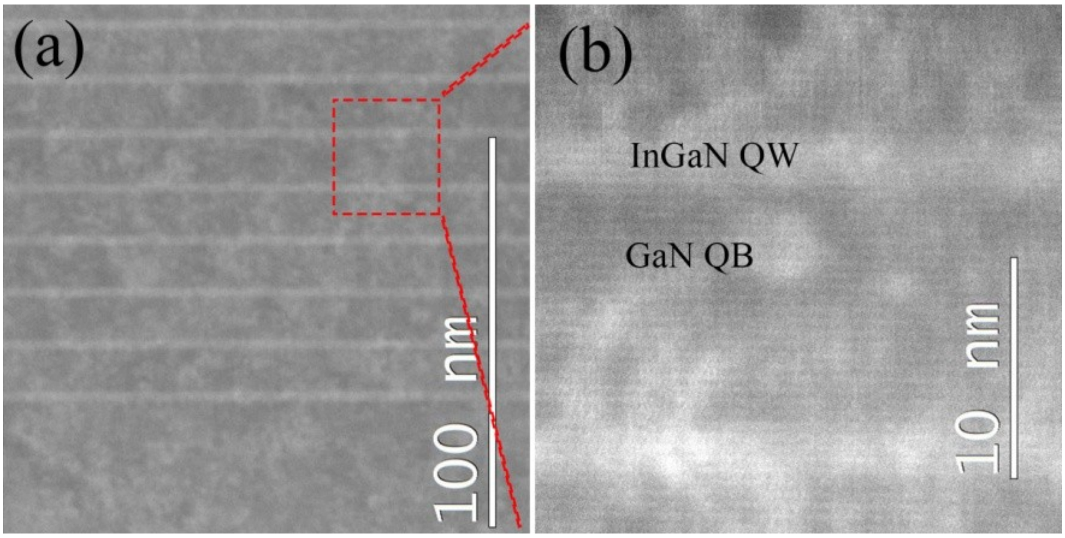

Figure S4. HAADF-STEM image of planar III-polar MQW samples with low magnification (**a**) and zoom-in view (**b**) of the individual QW and QB.

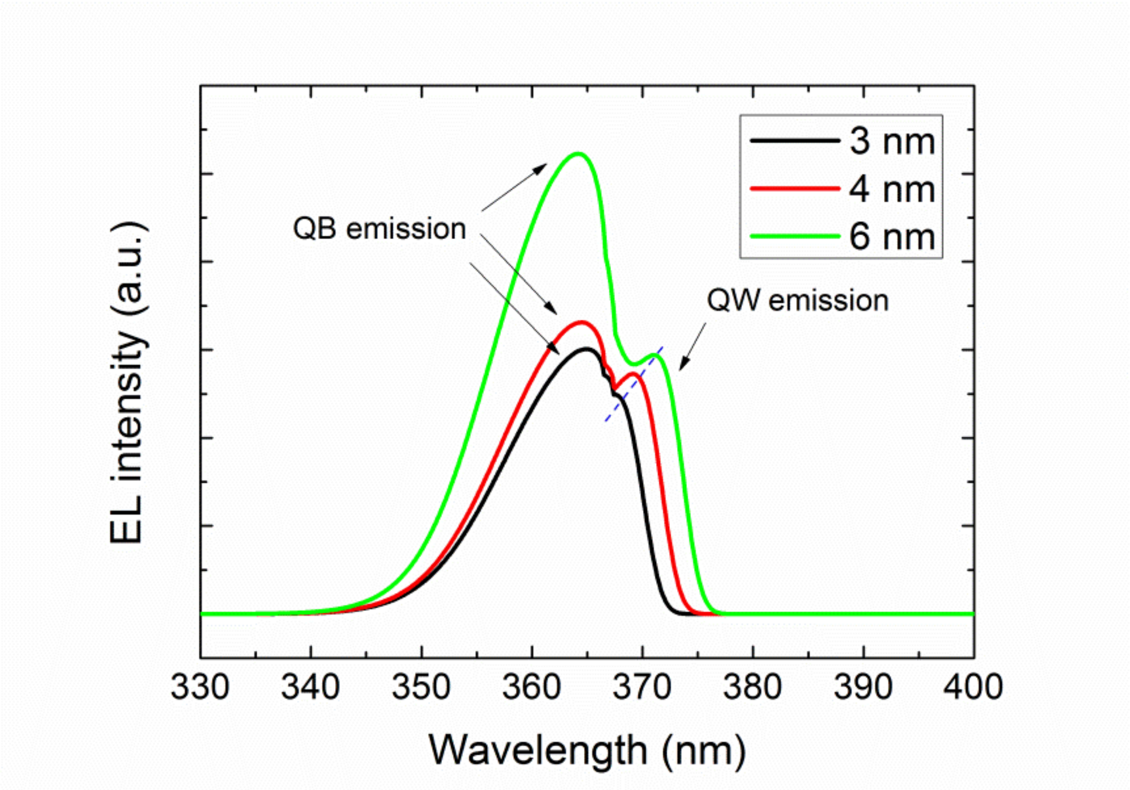

Figure S5. Simulated electroluminescence spectra of UVA-LED with $In_{0.03}Ga_{0.97}N$/GaN MQWs of varying QW thicknesses. A small red shift of less than 7 nm is observed when doubling the QW thickness.
